# Supplementary material for: Increased Serum Mir-150-3p Expression Is Associated with Radiological Lung Injury Improvement in Patients with COVID-19
Source: Viruses. 2022 Jun 23;14(7):1363. doi: 10.3390/v14071363 (PMC9323362; doi:10.3390/v14071363)
Supplement: Supplementary file 1 [file viruses-14-01363-s001.zip › viruses-1711656-supplementary.pdf]

Supplementary data

INCREASED SERUM MIR-150-3P EXPRESSION IS ASSOCIATED WITH RADIOLOGICAL LUNG INJURY IMPROVEMENT IN PATIENTS WITH COVID-19.

Larissa C.M. Bueno[1]; Layde R. Paim[1]; Eduarda O.Z. Minin[1]; Paulo R. Mendes[1]; Tatiana A. Kiyota[1]; Angelica Z. Schreiber[1], Bruna Bombassaro[2]; Eli Mansour[1]; Maria Luiza Moretti[1]; Jonathan Tak-Sum Chow[3]; Leonardo Salmena[3]; Otavio R. Coelho-Filho[1]; Licio A. Velloso[1,2]; Wilson Nadruz[1]; Roberto Schreiber[1].

Table S1- Patients included in the study according to radiological pulmonary status.

	Degree of involvement of the lung parenchyma (%)				
**Patient**	**Inclusion**	**Discharge**	**Radiological pulmonary status**	**Time between CT scans (days)**	**Supporting Medication**
P11	5-25	<5	improvement	5	Losartan/ iC1e/K
P19	50-75	26-49	improvement	6	Insulin
P10	50-75	5-25	improvement	12	Losartan/ icatibant
P26	5-25	<5	improvement	11	Dipyrone/Losartan/L-thyroxin/Clavulanic acid+Amoxicillin/Azithromycin
P27	26-49	5-25	improvement	8	iC1e/K
P25	50-75	26-49	improvement	21	iC1e/K
P24	>75	26-49	improvement	13	Dipyrone/Clavulanic acid+Amoxicillin/Azithromycin/Heparin/ iC1e/K
P23	50-75	26-49	improvement	13	Amoxicillin
P22	26-49	5-25	improvement	7	Dipyrone/ Clavulanic acid+Amoxicillin /Azithromycin/ iC1e/K
P30	50-75	26-49	improvement	7	Metformin/Insulin/ icatibant
P8	50-75	26-49	improvement	28	Dipyrone/Azithromycin/ iC1e/K
P18	50-75	5-25	improvement	22	Dipyrone/ Clavulanic acid+Amoxicillin /Azithromycin/Insulin/ iC1e/K

P28	50-75	26-49	improvement	19	Enalapril/Metformin/Insulin/icatibant
P9	5-25	<5	improvement	23	icatibant
P4	50-75	5-25	improvement	13	None
P7	26-49	5-25	improvement	15	Dipyrone/Azithromycin/Metformin
P1	26-49	26-49	without improvement	5	None
P12	50-75	50-75	without improvement	8	Dipyrone/Azithromycin/Insulin/Losartan/ iC1e/K
P2	5-25	5-25	without improvement	5	Dipyrone/icatibant
P13	5-25	5-25	without improvement	8	Dipyrone
P29	5-25	5-25	without improvement	11	Enalapril/Metformin/Insulin/icatibant
P6	5-25	5-25	without improvement	4	Dipyrone/icatibant
P16	5-25	26-49	without improvement	4	Dipyrone/ Clavulanic acid+Amoxicillin /Azithromycin/icatibant
P17	5-25	26-49	without improvement	5	Hydrochlorothiazide/Losartan
P21	5-25	26-49	without improvement	5	icatibant
P5	5-25	50-75	without improvement	28	Dipyrone/Azithromycin/icatibant
P20	5-25	26-49	without improvement	13	Dipyrone/ iC1e/K

iC1e/K - inhibitor of C1 esterase/kallikrein (Berinert®); icatibant (Firazyr®)

Table S2- Characteristics of participants at hospital admission according to radiological pulmonary improvement.

Parameter	With Improvement (n=16)	Without improvement (n=11)	p
Clinical characteristics			
Female/male	9/7	3/8	0.273
Age (years)	49.1 ± 13.4	53.6 ± 8.0	0.322
Body Mass Index (kg/m2)	33.2 ± 5.7	28.4 ± 6.5	0.094
Hypertension, n (%)	6 (37.5)	5 (45.5)	0.988
Diabetes mellitus, n (%)	6 (37.5)	4 (36.4)	0.952
Obesity, n (%)	12 (75)	7 (63.6)	0.836
Former smoker, n (%)	0 (0)	2 (18.2)	0.355
Systolic blood pressure (mmHg)	135.7 ± 19.2	128.1 ± 13.8	0.314
Diastolic blood pressure (mmHg)	83.1 ± 12.3	81.4 ± 18.7	0.778
Oxygen saturation (%)	89.5 ± 4.9	90.7 ± 2.0	0.471
PaO2/FiO2 ratio	240 [193-314]	289 [201-348]	0.367
Respiratory rate, per min	24.6 ± 5.7	22.1 ± 3.5	0.202
Symptoms onset before admission (days)	8.5 [6-10]	8.0 [7-9]	0.753
Admission at ward/ICU	8/8	7/4	0.759
O2 therapy	16	11	
ventilatory support	7 (44)	1 (9)	0.131
Laboratory data			
White cell count (x10^9/L)	7.8 [6.0-11.9]	6.0 [5.2-7.6]	0.134
Lymphocyte count (x10^9/L)	3.79 [1.34-13.52]	1.39 [0.95-2.22]	0.108
Platelet count (x10^9/L)	234.3 ± 113.4	209.0 ± 109.5	0.568
Plasma glucose (mg/dL)	151.6 ± 53.9	149.7 ± 85.9	0.948
Serum creatinine (mg/dL)	0.86 [0.70-0.90]	1.05 [0.84-1.08]	0.134
AST (U/L)	40 [27-49]	42 [33-67]	0.444
C-Reactive Protein (mg/L)	83.9 [43.2-148.0]	88.3 [42.3-132.0]	0.683
High-sensitivity troponin I, ng/L	9.1 [5.1-11.2]	7.0 [5.2-9.4]	0.474
Brain natriuretic peptide, ng/mL	63 [50-111]	178 [50-308]	0.141
D-dimer, µg/mL	732 [517-1054]	844 [542-1392]	0.610
Urea (mg/dL)	27 [20-37]	41 [32-42]	0.064
Global lung CT scoring			
Score	17.5 ± 4.9	13.7 ± 3.6	0.040
Evolution			
Supplementary oxygen, n (%)	16 (100)	11 (100)	1.000
Mechanical ventilation, n (%)	7 (44)	1 (9)	0.131

Abbreviations: AST, aspartate aminotransferase; ICU – intensive care unit. Patient data were compared using Student's t test and Mann-Whitney test for continuous variables with normal or non-normal distribution, respectively. The chi-square test was used to assess frequency differences between groups. $p < 0.05$ was considered statistically significant.

Table S3- MiRNA binding sites on SARS-CoV-2 receptors predicted by the RNA22 tool.

	receptors	Binding sites	p-value
mir-150-3p	TMPRSS	3	0.131-0.389
miR-191-5p	ACE2	1	0.123

ACE2 (NCBI Reference Sequence: NM_001371415.1)
TMPRSS (NCBI Reference Sequence: NM_001135099.1)

Table S4- MiRNA binding sites in the SARS-CoV-2 genome predicted by the RNA22 tool

<table>
<tr><th></th><th>Position of predict target</th><th>Gene of predict target</th><th>Heteroduplex</th><th>p-value</th></tr>
<tr><td>mir-150-3P</td><td>9290</td><td>nsp4</td><td>GATTATTACAGATCTTTACCAG
: ||| || ||||||
GACAGGGGGTCCGGACATGGTC</td><td>0.044</td></tr>
<tr><td></td><td>28940</td><td>N</td><td>CTG-CTTGACAG-ATTGAACCAG
|||| |:: ||| :|| |||||
GACAGGG-GGTCCGGACATGGTC</td><td>0.046</td></tr>
<tr><td></td><td>26009</td><td>ORF3a</td><td>ACTTCACTTCAGACTATTACCAG
|| |::||| |: ||||||
GACAG-GGGGTCCGGACATGGTC</td><td>0.050</td></tr>
<tr><td></td><td>16941</td><td>nsp13</td><td>AAGTGCACCTACACTAGTGCCAC
|| | ||:| |: ||:|||
GACAGG-GGGTCCGGACATGGTC</td><td>0.080</td></tr>
<tr><td></td><td>19667</td><td>nsp15</td><td>ATGGACAACAGGGTGAAGTACCAG
|| | |||| : |||||||
GACAGGGGGTCCGG--ACATGGTC</td><td>0.147</td></tr>
<tr><td></td><td>26621</td><td>M</td><td>TCTTCTACAATTTGCCTATGCCAA
|| | |||| |:|||
GACAGGGG--GTCCGGACATGGTC</td><td>0.157</td></tr>
<tr><td></td><td>27611</td><td>ORF7a</td><td>ACGTCTATCAGTTACGTGCCAG
|||: ||| ||:||||
GACAGGGGGTCCGGACATGGTC</td><td>0.380</td></tr>
<tr><td>miR-212-3p</td><td>16840</td><td>nsp13</td><td>AAAGGTGACTATGGTGATGCTGTTG
|||||| || | :|||||:
CCGGCACTGA--CCTC--TGACAAT</td><td>0.303</td></tr>
<tr><td></td><td>18319</td><td>nsp14</td><td>TGTCATGCTACTAGAGAAGCTGTTG
|:| || ||| ||| :|||||:
CCGGCAC--TGACCTC--TGACAAT</td><td>0.340</td></tr>
<tr><td>miR-191-5p</td><td>16841</td><td>nsp13</td><td>AAGGTGACTATGGTGATGCTGTTG
|| || ||| ||| |:||||
GTCGACGAAAACC-CTAAGGCAAC</td><td>0.303</td></tr>
<tr><td>miR-151a-3p</td><td>23603</td><td>S</td><td>CCTCGGCGGGCACGTAGTGTAG
||||:: |:|| :||| |||
GGAGTTCCTCG-AAGTCAGATC</td><td>0.126</td></tr>
<tr><td></td><td>28960</td><td>N</td><td>GCTTGAG-AGCAAAATGTCTGG
|| || ||| ||||:|
GGAGTTCCTCG-AAGTCAGATC</td><td>0.046</td></tr>
<tr><td>miR-92a-3p</td><td>13169</td><td>nsp10</td><td>ACTGGTACTGGTCA-G-GCAATA
|| ||: || || | ||||||
TGTCCG-GCCCTGTTCACGTTAT</td><td>0.218</td></tr>
</table>

The p-value represents the likelihood that the target site loci is random. That is, a lower p-value represents a greater chance that the loci contain a valid MRE.

Table S5- Correlation analysis between miRNA expression by OpenArray with the laboratory tests 30 days after hospital discharge.

	Log miR-150-3p		Log miR-92a-3p		Log miR-151a-3p		Log miR-191-5p		Log miR-548a-3p		Log miR-548c-3p		Log miR-212-3p	
	r	p	r	p	r	p	r	p	r	p	r	p	r	p
White cell count	0.148	0.480	0.243	0.221	0.141	0.564	0.124	0.547	-0.068	0.759	-0.174	0.417	-0.475	0.054
Lymphocyte count	0.210	0.336	0.266	0.198	-0.018	0.944	-0.620	**0.001**	-0.543	**0.009**	-0.548	**0.007**	-0.288	0.279
Platelet count	0.098	0.642	-0.107	0.596	-0.083	0.735	-0.046	0.823	-0.002	0.993	-0.332	0.113	-0.103	0.694
Plasma glucose	0.116	0.608	0.190	0.373	0.028	0.918	0.158	0.471	0.119	0.606	-0.035	0.880	-0.191	0.513
Serum creatinine	-0.270	0.192	-0.360	0.065	0.219	0.369	-0.392	**0.048**	0.463	**0.026**	0.352	0.092	0.091	0.729
AST	0.069	0.742	-0.135	0.502	-0.274	0.257	-0.249	0.220	0.276	0.203	0.157	0.465	0.342	0.179
C-Reactive Protein	-0.099	0.645	0.152	0.458	0.244	0.315	0.073	0.728	0.050	0.824	-0.018	0.936	-0.368	0.161
Hs troponin I	-0.023	0.915	0.104	0.614	0.028	0.910	-0.204	0.328	0.206	0.357	0.226	0.300	-0.221	0.412
Brain natriuretic peptide	-0.330	0.144	0.041	0.852	0.324	0.189	-0.269	0.226	0.138	0.574	0.255	0.265	-0.017	0.956
D-dimer	-0.182	0.395	-0.205	0.314	0.203	0.404	-0.160	0.445	0.394	0.070	0.479	**0.021**	-0.106	0.696
Urea	-0.165	0.430	-0.151	0.453	0.146	0.550	-0.242	0.233	0.341	0.111	0.184	0.390	-0.016	0.951

The correlation of log-transformed expression of miRNAs with laboratory tests was assessed by Spearman's Method. $p < 0.05$ was considered statistically significant.

Table S6-Correlation analysis between miRNA expression at admission with the laboratory tests.

	Log miR-150-3p (admission)	
	r	p
White cell count (x109/L)	0.230	0.257
Lymphocyte count (x109/L)	-0.602	**0.002**
Platelet count (x109/L)	0.411	**0.037**
Plasma glucose (mg/dL)	0.096	0.664
Serum creatinine (mg/dL)	0.211	0.300
AST (U/L)	-0.035	0.864
C-Reactive Protein (mg/L)	-0.255	0.218
High-sensitivity troponin I, ng/L	0.135	0.520
Brain natriuretic peptide, ng/mL	0.115	0.610
D-dimer, µg/mL	0.028	0.893
Urea (mg/dL)	0.256	0.208

The correlation of log-transformed expression of miRNAs with laboratory tests was assessed by Spearman's Method. $p < 0.05$ was considered statistically significant.
